# IKKγ-Mimetic Peptides Block the Resistance to Apoptosis Associated with Kaposi's Sarcoma-Associated Herpesvirus Infection

**DOI:** 10.1128/JVI.01170-17

**Published:** 2017-11-14

**Authors:** Louise C. Briggs, A. W. Edith Chan, Christopher A. Davis, Nicholas Whitelock, Hajira A. Hotiana, Mehdi Baratchian, Claire Bagnéris, David L. Selwood, Mary K. Collins, Tracey E. Barrett

**Affiliations:** a Institute for Structural and Molecular Biology, Department of Biological Sciences, Birkbeck College, London, United Kingdom; b The Wolfson Institute for Biomedical Research, University College London, London, United Kingdom; c MRC-University of Glasgow Centre for Virus Research, Glasgow, Scotland, United Kingdom; d Biopharmaceutical Bioprocessing Technology Centre, Newcastle University, Newcastle upon Tyne, United Kingdom; e The Medical Research Council Centre for Medical Molecular Virology, Division of Infection and Immunity, University College London, London, United Kingdom, and Division of Advanced Therapies, National Institute of Biological Standards and Control, South Mimms, Potters Bar, Herts, United Kingdom; f Okinawa Institute of Science and Technology Graduate University, Okinawa, Japan; University of Southern California

**Keywords:** Kaposi's sarcoma-associated herpesvirus, antimicrobial peptides, vFLIP

## Abstract

Primary effusion lymphoma (PEL) is a lymphogenic disorder associated with Kaposi's sarcoma-associated herpesvirus (KSHV) infection. Key to the survival and proliferation of PEL is the canonical NF-κB pathway, which becomes constitutively activated following overexpression of the viral oncoprotein KSHV vFLIP (ks-vFLIP). This arises from its capacity to form a complex with the modulatory subunit of the IκB kinase (IKK) kinase, IKKγ (or NEMO), resulting in the overproduction of proteins that promote cellular survival and prevent apoptosis, both of which are important drivers of tumorigenesis. Using a combination of cell-based and biophysical assays together with structural techniques, we showed that the observed resistance to cell death is largely independent of autophagy or major death receptor signaling pathways and demonstrated that direct targeting of the ks-vFLIP–IKKγ interaction both in cells and *in vitro* can be achieved using IKKγ-mimetic peptides. Our results further reveal that these peptides not only induce cell killing but also potently sensitize PEL to the proapoptotic agents tumor necrosis factor alpha and etoposide and are the first to confirm ks-vFLIP as a tractable target for the treatment of PEL and related disorders.

**IMPORTANCE** KSHV vFLIP (ks-vFLIP) has been shown to have a crucial role in cellular transformation, in which it is vital for the survival and proliferation of primary effusion lymphoma (PEL), an aggressive malignancy associated with infection that is resistant to the majority of chemotherapeutic drugs. It operates via subversion of the canonical NF-κB pathway, which requires a physical interaction between ks-vFLIP and the IKK kinase modulatory subunit IKKγ. While this interaction has been directly linked to protection against apoptosis, it is unclear whether the suppression of other cell death pathways implicated in ks-vFLIP pathogenesis is an additional contributor. We demonstrate that the interaction between ks-vFLIP and IKKγ is pivotal in conferring resistance to apoptosis. Additionally, we show that the ks-vFLIP–IKKγ complex can be disrupted using peptides leading to direct killing and the sensitization of PEL cells to proapoptotic agents. Our studies thus provide a framework for future therapeutic interventions.

## INTRODUCTION

Kaposi's sarcoma-associated herpesvirus (KSHV) is the most common cause of malignancy among AIDS patients (1, 2). In addition to being the main etiological agent of Kaposi's sarcoma (KS), KSHV infection has also been linked to primary effusion lymphoma (PEL) and multicentric Castleman's disease (3, 4). While AIDS-KS can be successfully treated with antiretroviral drugs at the early stages of onset, there are to date no highly effective treatments for the advanced disease or associated lymphoproliferative disorders. In particular, PEL has an extremely poor prognosis for which average survival rates vary between 3 and 6 months following diagnosis, prompting an urgent need for the identification of novel drug targets and therapeutics. During the latent phase of infection, KSHV produces a limited number of viral proteins that are essential for subverting the host to promote survival, immune evasion, viral replication, and propagation (5). One such protein is KSHV vFLIP (ks-vFLIP), a cellular FLIP homologue that targets the canonical and alternative NF-κB transcriptional pathways, rendering them constitutively active (6, 7). This persistent activation results in the deregulated overexpression of gene products known to promote cellular survival and inhibit apoptosis, both of which have been strongly implicated in transformation. It has also been demonstrated that ks-vFLIP is crucial for the proliferation of PEL (8, 9), promoting anchorage-independent survival of endothelial cells (10) while suppressing autophagy (11) and death receptor signaling (8, 12).

Although persistent activation of both the canonical and alternative NF-κB pathways by KSHV have been documented, its effects on the canonical pathway have been the most extensively characterized. Key to activation are the p65(RelA)/p50 or p65/c-Rel heterodimeric transcription factors (13–15). These are normally sequestered in the cytoplasm as a consequence of their associations with the IκB family of inhibitory proteins that occlude their nuclear localization sequences, thus preventing transit to the nucleus. On activation, the IκB proteins are targeted for degradation by the 26S proteosome following phosphorylation and subsequent K48-linked ubiquitination. This culminates in the liberated transcription factors being able to enter the nucleus and initiate transcription of the various genes under their control. Central to this process is the IκB kinase (IKK) kinase complex or signalosome (16) that instigates the initial phosphorylation of the IκB proteins. The core IKK complex consists of the kinase subunits IKKα and IKKβ together with the essential modulatory element IKKγ (also referred to as NEMO), which is the target for ks-vFLIP. By means of a physical interaction with IKKγ, ks-vFLIP is able to “lock” the IKK kinase complex into a persistently phosphorylated state via a mechanism that appears to be independent of known upstream cellular activators, such as Traf2, Traf3, Traf6, LUBAC, Tak-1, and HOIL-1 (17), and is thus distinct to those resulting from stimulation by endogenous proinflammatory cytokines or cellular FLIP variants (18).

In previous studies, the crystal structure of ks-vFLIP bound to IKKγ (PDB code 3CL3) was determined (19). Pivotal to complex formation are amino acids within the region of IKKγ spanning residues 230 to 248 (a dimeric coiled-coil) that are accommodated within two clefts of a ks-vFLIP monomer. Significantly, disruption of important interacting residues within this sequence using site-directed mutagenesis has been shown to drastically reduce the levels of NF-κB activation. Here we report that a conformationally constrained, stapled peptide based on this interacting motif (spIKKγ) and an analogous glutathione *S*-transferase (GST)-peptide fusion protein (GST-IKKγ) not only are competitive inhibitors of IKKγ *in vitro* but also, when combined with etoposide (ETO) or tumor necrosis factor alpha (TNF-α), drastically reduce the viability of BC3 and BCP-1 cells. This is a direct consequence of removing the apoptotic block imposed by the ks-vFLIP-mediated overproduction of prosurvival/antiapoptotic proteins, in which there appears to be little coinvolvement of ATG3-dependent autophagic and no involvement of important death receptor-mediated pathways. Our findings not only demonstrate ks-vFLIP as an amenable target for the treatment of PEL and other KSHV-related malignancies using gene therapies but also suggest that stapled peptides/helical mimetics could form a basis for the design of future high-potency therapeutics.

## RESULTS

### The antiapoptotic effects of ks-vFLIP overexpression are largely independent of autophagic and key death receptor signaling pathways.

**FIG 1:**
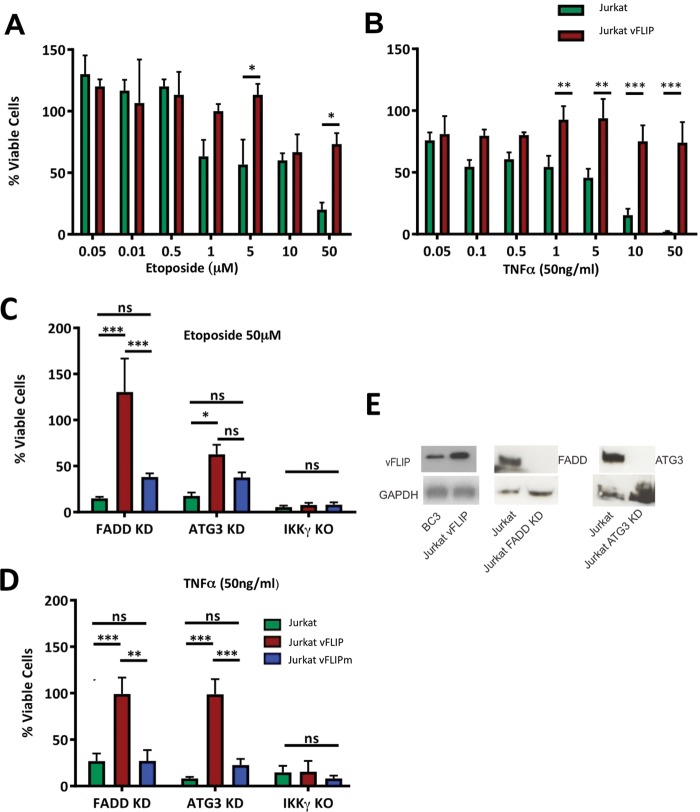
Antiapoptotic effects of ks-vFLIP overexpression in Jurkat cells. Viability assays were conducted with Jurkat cells treated with various concentrations of ETO (A) and TNF-α (B). The ability of ks-vFLIP to protect Jurkat cells against the effects of ETO (C) and TNF-α (D) where FADD and ATG3 have been silenced using siRNA, together with those null for IKKγ, was assessed. (E) (Left image) Immunoblot comparing ks-vFLIP levels in Jurkat vFLIP cells with those in the KSHV-transformed PEL cell line BC3. (Right and middle images) Downregulation of FADD and ATG3 in Jurkat cells using siRNA. Asterisks indicate significant differences (*, *P* < 0.05, **, *P* < 0.01, ***, *P* < 0.001; z test). ns, not significant.

Although it has been established that dysregulated activation of the canonical NF-κB pathway by ks-vFLIP renders mouse embryonic fibroblasts resistant to apoptosis induced by TNF-α (12), we sought to ascertain whether ks-vFLIP expression also prevents cell death induced by ETO. Unlike TNF-α, which instigates death receptor signaling, ETO, a topoisomerase II inhibitor, initiates cell death through the generation of DNA double-strand breaks (20). Our results revealed that at the highest concentration of ETO used (50 μM), the survival of Jurkat cells was only moderately reduced compared to that of cells null for ks-vFLIP, which were severely compromised (Fig. 1A). The results with ETO closely mirror the trend observed for those treated with TNF-α (Fig. 1B) and were consistent with those of Tolani et al. (12), thus confirming ks-vFLIP as a key factor in the inhibition of apoptosis able to override the effects of both ETO and TNF-α. We next investigated the potential involvement of death-inducing signaling complex (DISC) formation and autophagy since ks-vFLIP has been cited (somewhat controversially) to interact with procaspase 8, an effector caspase whose activation requires the adaptor protein FADD (21), and to physically associate with the ubiquitin-like conjugating enzyme ATG3, which is essential for the formation of autophagosomes (11). Viability assays were therefore conducted using Jurkat cells in which FADD and ATG3 were silenced (using small interfering RNA [siRNA]) together with those null for IKKγ in the presence of both ETO and TNF-α (Fig. 1C and D). Figure 1E shows that the level of expression of ks-vFLIP in the engineered Jurkat cells is 3- to 5-fold greater than observed in the KSHV-transformed PEL cell line BC3 and that both FADD and ATG3 are undetectable by immunoblotting in those treated with siRNA. While cell survival was severely compromised in all three scenarios, it could to a large extent be rescued in the FADD/ATG3 siRNA Jurkat cells overexpressing wild-type (WT) ks-vFLIP, although to a lesser degree in the case of ATG3 and ETO. Only cells null for IKKγ were completely refractory to the protective effects of ks-vFLIP. Furthermore, the A57L ks-vFLIP mutant, which has been shown to disrupt IKKγ binding (19), could not protect cells against apoptosis induced by ETO or TNF-α (Fig. 1C and D), highlighting the importance of the ks-vFLIP–IKKγ interaction.

### Targeting the ks-vFLIP–IKKγ interaction and design of the IKKγ stapled peptide spIKKγ.

**FIG 2:**
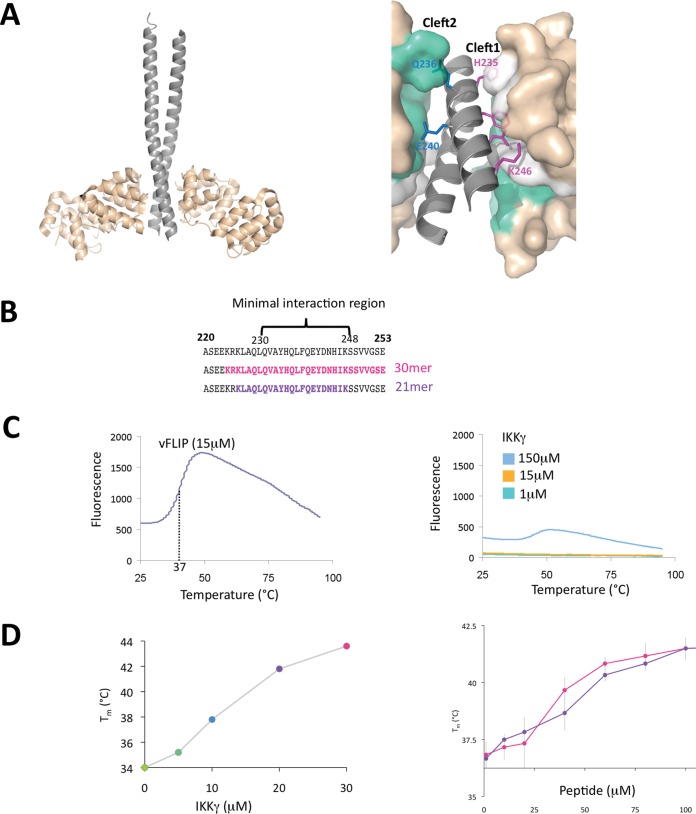
vFLIP-IKKγ interaction and peptide thermofluor analysis. (A) (Left) Cartoon depiction of the ks-vFLIP–IKKγ heterotetramic complex (PDB code 3CL3). The IKKγ and ks-vFLIP monomers are shown in gray and wheat, respectively. (Right) The ks-vFLIP-IKKγ dimer interface highlighting cleft 1 (white) and cleft 2 (cyan). Residues essential for the formation of the cleft 1 interactions (H235, F238, D242, and K246) are colored magenta, and those that interact with cleft 2 (Q236 and E240) are colored blue. (B) IKKγ ks-vFLIP minimal interacting motif (residues 230 to 248) together with those forming the 30- and 21-mer peptides (pink and violet, respectively) used in the thermofluor studies. (C) Thermal denaturation profiles for ks-vFLIP (left) and IKKγ (right) at various concentrations. The lack of a significant response over the range of concentrations tested for IKKγ is owing to its dimeric coiled configuration that lacks a substantial hydrophobic core. (D) Thermal denaturation of ks-vFLIP in the presence of various concentrations of IKKγ (left) and the 21-mer (pink) and 30-mer (violet) (right) peptides.

Having ascertained that neither DISC formation or autophagy is a major factor in conferring ks-vFLIP-induced resistance to apoptosis, we next investigated whether the ks-vFLIP–IKKγ interaction, shown to be essential, could be successfully disrupted by mimetic peptides *in vitro*. The original ks-vFLIP–IKKγ structure (PDB code 3CL3) adopts a heterotetrameric configuration in which a ks-vFLIP dimer associates with IKKγ in a dimeric, parallel, helical coiled-coil conformation (Fig. 2A). Although complex formation involves two distinct regions of ks-vFLIP (cleft 1 and cleft 2) simultaneously bound to each IKKγ monomer in the dimeric assembly (Fig. 2A), it was shown that the protein-protein interactions mediated by cleft 1 are crucial (19). We therefore sought to establish whether IKKγ binding to ks-vFLIP could be mimicked by single peptides incorporating residues shown to mediate important cleft 1 interactions. To achieve this, an unmodified 30-mer peptide (residues 224 to 253) was designed to encompass the interacting motif and neighboring residues along with a 21-mer (residues 226 to 248), only marginally longer than the motif itself (residues 230 to 248 [Fig. 2B]). These were commercially synthesized and their ability to associate with ks-vFLIP was assessed using a thermofluor assay (Fig. 2C and D). While neither the 21- or 30-mer peptide could emulate the ∼8°C shifts in melting temperature observed with the IKKγ fragment encompassing residues 150 to 272 [IKKγ(150–272)] used in the original structural studies at a concentration of 30 μM (contributions from denaturation of the IKKγ dimer being insignificant owing to its lack of a substantial hydrophobic core [Fig. 2C]), both gave rise to shifts of ∼4°C at 100 μM despite being of significantly different lengths. These results were the first indication that ks-vFLIP could be targeted using single peptides centered on the minimal interacting motif.

The fact that we were able to at least partially substitute IKKγ for unmodified peptides led us to investigate whether the IKKγ ks-vFLIP minimal interacting motif could be engineered into a conformationally constrained, helical peptide through incorporation of an appropriate aliphatic linker or “staple” (22). This strategy has been successfully used in several distinct systems involving protein-protein interactions mediated through helical recognition (23–26). It relies on the premise that one of the interacting partners can be simulated by a helical peptide resulting from the replacement of two nonessential amino acids by (*S*)-2-(2′ pentenyl)alanine that are subsequently cyclized to produce a constraining aliphatic linker. Such peptides have been frequently found to have enhanced affinity for their targets as well as innate cell permeability, thought to partially derive from the hydrophobic nature of the linker and favored by an overall net positive charge.

**FIG 3:**
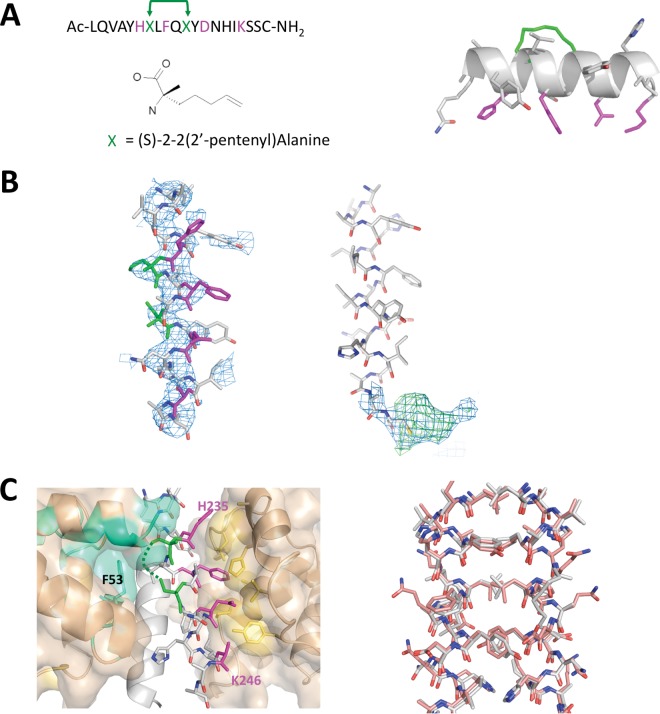
spIKKγ and the crystal structure of the GB1–ks-vFLIP–spIKKγ complex. (A) (Left) Sequence of the *in silico*-designed IKKγ-mimetic peptide (spIKKγ), together with the position and structure of (*S*)-2-(2′ pentenyl)alanine used to replace Q236 and E240 (green). The key cleft 1 binding residues are depicted in magenta. (Right) Energy-minimized model of spIKKγ highlighting the aliphatic linker (green) after cyclization and its position relative to the cleft 1 binding residues on the opposite face of the helix. (B) (Left) Refined coordinates of spIKKγ superimposed on the initial 2mFo-DFc electron density map (blue, contoured at 1σ), calculated using the coordinates of ks-vFLIP from PDB entry 3CL3 alone after molecular replacement. (Right) 2mFo-DFc (blue, contoured at 1σ) and mFo-DFc (green, contoured at 3σ) electron densities associated with the C-terminal cysteine of one spIKKγ peptide. (C) (Left) ks-vFLIP–spIKKγ dimer interface (for clarity, one IKKγ monomer is depicted as a cartoon [light gray]). The cleft 1 interactions (magenta for spIKKγ and yellow-orange for cleft 1 in ks-vFLIP) are entirely conserved. The aliphatic staple (green), though partially ordered, projects into the cavity between cleft 1 and cleft 2 (cyan) of the opposite ks-vFLIP monomer alongside F53. (Right) spIKKγ-spIKKγ dimer (gray) superimposed on the analogous interface in the ks-vFLIP-IKKγ(150–272) complex (pink) shows an almost identical configuration and arrangement of residues.

Inspection of the ks-vFLIP–IKKγ structure identified Q236 and E240 as potential sites on IKKγ for an i, i + 4 staple given that they are nonessential (Fig. 3A). To test our rationale *in silico*, a model peptide was constructed of the IKKγ minimal interacting motif (L230 to S248) incorporating the staple and energy minimized (MMFF94x force field) using the Molecular Operating Environment (MOE) software package (2014.09; Chemical Computing Group Inc., Montreal, QC, Canada). Following minimization, the helical geometry and overall configuration of the peptide were observed to remain unchanged. The model was then docked into cleft 1 of ks-vFLIP and the complex subjected to energy minimization in which the position of the protein moiety was fixed while permitting movement of the peptide. The resulting energy-minimized complex revealed that all of the important interactions remained unperturbed. Based on these findings, the modified peptide (spIKKγ [Fig. 3A]) was subsequently synthesized with an added C-terminal cysteine to permit future conjugation to cell transporter molecules or fluorophores. Inclusion of this nonnative residue had no effect on the protein-peptide interactions as initially judged by *in silico* modeling and later confirmed by *in vitro* studies (see below).

### spIKKγ is an effective mimetic of IKKγ.

Our focus on a single peptide together with the presence of the aliphatic staple made it difficult to predict the exact nature of a ks-vFLIP–spIKKγ complex and, in particular, its stoichiometry. To address this, we cocrystallized ks-vFLIP with spIKKγ. Similar to the ks-vFLIP–IKKγ heterotetramic complex (PDB code 3CL3), two molecules of GB1–ks-vFLIP were seen to comprise the asymmetric unit, where further analysis revealed clear electron density for peptides bound to each ks-vFLIP monomer (Fig. 3B). Unexpectedly, sizeable density associated with the C-terminal cysteines of the two peptides could also be identified. Based on size and overall shape, it is likely to originate from the vitamin B_12_ additive, but owing to disorder, it could not be unambiguously modeled for either monomer (Fig. 3B).

The overall configuration of the ks-vFLIP–spIKKγ complex revealed a heterotetrameric arrangement analogous to that observed in the ks-vFLIP-IKKγ(150–272) structure, where both peptides contacted clefts 1 and 2 (Fig. 3C). As indicated by our modeling studies, the ks-vFLIP–spIKKγ binding interface mediated by cleft 1 showed a high degree of structural similarity to the original ks-vFLIP–IKKγ(150–272) crystal structure. Crucially, the H235, F238, E242, and K246 quartet, central to binding and subsequent NF-κB activation, form similar contacts with the cleft 1 region in the peptide complex. Also in common with the ks-vFLIP–IKKγ(150–272) heterotetramer, there were few specific interactions between spIKKγ and cleft 2 (Fig. 3C). Notably, however, the aliphatic staples replacing the side chain moieties of Q236 and E240 (though only partially ordered) pack alongside the aromatic side chain of F53 located within the interface between clefts 1 and 2, where they form van Der Waals interactions. The high level of conservation between ks-vFLIP–spIKKγ and ks-vFLIP–IKKγ(150–272) extends beyond the ks-vFLIP binding region to the peptide-peptide interface, which adopts a very similar composition and configuration to the IKKγ dimer evident in the complex with PDB code 3CL3 (Fig. 3C). This arises from the formation of a nearly identical noncrystallographic dimer.

**FIG 4:**
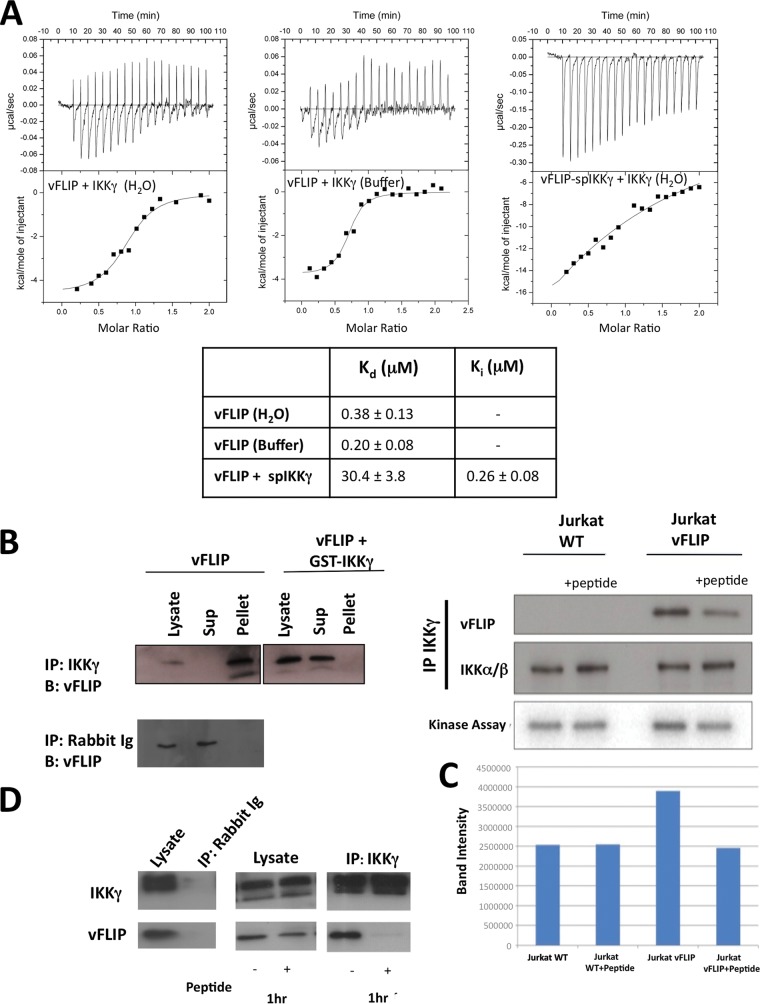
ITC experiments and the effects of IKKγ-mimetic peptides on NF-κB activation. (A) (Top) ITC data obtained for the following: IKKγ titrated into GB1–ks-vFLIP in which both proteins were dialyzed into deionized water (left), an analogous experiment but with the proteins dialyzed into buffer (see Materials and Methods) (middle), and a competition assay in which spIKKγ was incubated with GB1–ks-vFLIP prior to titration of IKKγ (performed in deionized water) (right). (Bottom) Table showing the corresponding “apparent” *K_d_*s and *K_i_* for spIKKγ. (B) Immunoprecipitation assays involving Jurkat cell lysates in which ks-vFLIP was expressed in the presence and absence of GST-IKKγ using anti-IKKγ antiserum (upper image) and normal rabbit serum (lower image). GST-IKKγ prevents the association of ks-vFLIP with IKKγ, evidenced by the absence of ks-vFLIP from the pellet fraction. (C) (Top) Immunoprecipitation trials using Jurkat cell lysates treated with 50 μM spIKKγ in which ks-vFLIP was absent or overexpressed. (Bottom) Results of a kinase assay measuring the levels of phosphorylated IκB in the presence and absence of spIKKγ. (D) Similar to panel C except that immunoprecipitation assays were performed using BC3 cell lysates. The rabbit antiserum control is shown in the leftmost image.

We next sought to assess the affinity of spIKKγ for vFLIP using isothermal calorimetry (ITC). Owing to poor solubility in aqueous solutions, a competition assay was performed in which IKKγ was titrated into GB1–ks-vFLIP preincubated with spIKKγ (Fig. 4A). In keeping with a high affinity, the apparent dissociation constant (*K_d_*) of the vFLIP-IKKγ interaction in the presence of peptide increased approximately 80-fold, from 380 nM to 30 μM. This corresponds to a *K_i_* of 257 nM and is thus comparable to that of IKKγ(150–272).

### GST-IKKγ and spIKKγ inhibit ks-vFLIP-induced NF-κB activation.

Having established that peptides can mimic IKKγ on the basis of our thermofluor, structural, and ITC studies, we next investigated their ability to attenuate NF-κB activation. For cellular expression, a GST–IKKγ–20-mer fusion protein (comprising the exact amino acid sequence of spIKKγ including the nonnative cysteine) was constructed, coexpressed with ks-vFLIP in Jurkat cells, and probed for its capacity to disrupt assembly of the ks-vFLIP–IKKγ complex (detected by immunoprecipitation using anti-IKKγ antibodies). Remarkably, the GST-peptide fusion protein virtually abolished formation of the ks-vFLIP–IKKγ complex (Fig. 4B), demonstrating that peptides have the potential to severely attenuate NF-κB activation in cells. The ability of spIKKγ to inhibit the ks-vFLIP–IKKγ interaction in cell extracts was next assessed by treating Jurkat cell lysates with spIKKγ for 1 h at 4°C, followed by immunoprecipitation of IKKγ and detection of any associated ks-vFLIP (Fig. 4C). In parallel, IKK kinase activity in the immunoprecipitate (and hence NF-κB activation) was also assessed by quantitatively measuring the levels of phosphorylation of added recombinant IκB (Fig. 4C). Our results revealed that the quantities of bound ks-vFLIP detected were substantially reduced in lysates treated with peptide. This trend was observed in the kinase assays, which surprisingly showed that in the presence of spIKKγ, ks-vFLIP-induced activation is reduced back to background levels. Similar results were also obtained when experiments were repeated with lysates from the PEL cell line BC3, in which ks-vFLIP is endogenously expressed (Fig. 4D). Our findings demonstrate that spIKKγ can dissociate preformed ks-vFLIP–IKKγ complexes, leading to the near abolition of induced NF-κB activation.

### Both GST-IKKγ and spIKKγ promote PEL cell killing, particularly in the presence of TNF-α and ETO.

**FIG 5:**
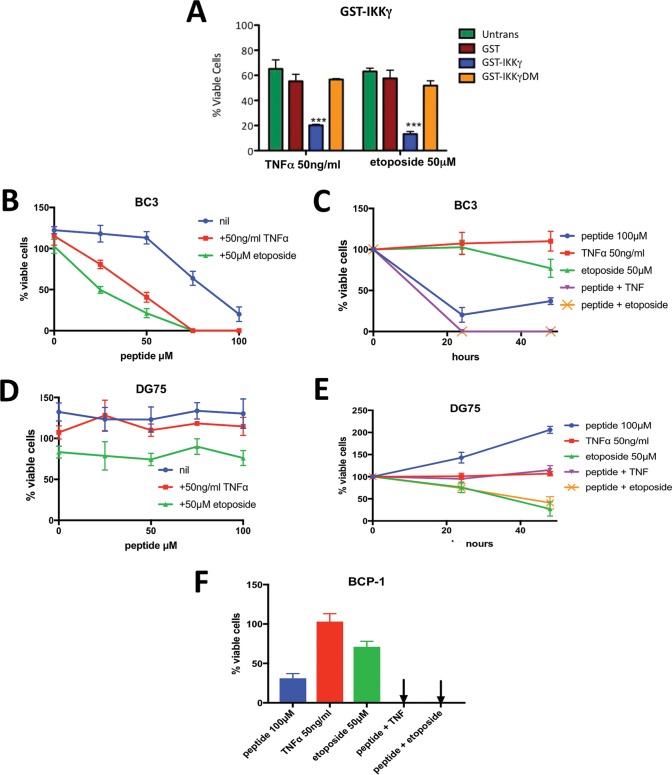
Capacity of IKKγ-mimetic peptides to kill BC3 and BCP-1 cells in the presence and absence of TNF-α and ETO. (A) Viability assays using BC3 cells coexpressing GST-IKKγ (or GST-IKKγDM harboring the F238A D242R double mutant, known to impair both vFLIP binding and NF-κB activation) and ks-vFLIP alone or following treatment with TNF-α or ETO. (B) Viability assays in which BC3 cells expressing ks-vFLIP were treated with various concentrations of spIKKγ in the presence and absence of TNF-α or ETO at the specified concentrations. (C) Assays in which the viability of BC3 cells treated with spIKKγ, TNF-α, ETO, or combinations of spIKKγ and TNF-α or ETO (at the specified concentrations) were monitored after 24 and 48 h. The curves for spIKKγ plus TNF-α (magenta) and spIKKγ plus ETO (yellow) overlap. (D) Effects of TNF-α and ETO (at the specified concentrations) on the viability of DG75 cells at various concentrations of spIKKγ. (E) Control experiment illustrating the individual effects of spIKKγ, TNF-α, and ETO together with the spIKKγ-plus-TNF-α and spIKKγ-plus-ETO combinations on the viability of DG75 cells (at the specified concentrations) at 24 and 48 h. While spIKKγ and TNF-α failed to have a negative impact (either alone or in combination), a significant reduction in viability was observed for cells treated with ETO (or ETO plus spIKKγ) at 48 h. (F) Viability assays conducted with BCP-1 cells similar to those for panel B but performed at the indicated concentrations of spIKKγ, TNF-α, and ETO.

Given their profound impact on NF-κB activation, we next assessed whether IKKγ-mimetic peptides could sensitize BC3 cells to TNF-α and ETO and thus stimulate cell death. To investigate this *in cellulo*, BC3 cells were transduced with the GST-IKKγ peptide vector prior to treatment with TNF-α or ETO, and after 24 h, their viabilities were assessed. As a control, similar trials were conducted but with GST-IKKγDM, which harbors the same peptide containing the F238R D242R IKKγ double mutations previously shown to impair ks-vFLIP binding and consequently NF-κB activation (27). The combination of GST-IKKγ and either TNF-α or ETO significantly reduced cell viability compared to those with GST-IKKγDM and GST (Fig. 5A), consistent with removal of the ks-vFLIP-induced apoptotic block. We also determined whether spIKKγ could have a similar effect. Owing to the presence of the aliphatic linker and overall positive charge (+0.1), we reasoned that spIKKγ was likely to be cell permeable based on studies involving a diverse range of unrelated stapled peptides (28–32). BC3 cells were therefore treated with spIKKγ either alone or in combination with TNF-α or ETO (Fig. 5B) and their viabilities assessed after 24 h. spIKKγ at the higher concentrations tested caused a significant reduction in their viability (50% inhibitory concentration [IC_50_] ∼ 75 μM after 24 h). Either TNF-α or ETO caused a striking enhancement of spIKKγ-induced BC3 cell death (Fig. 5B and C), but showed negligible effects individually. When viability was assessed at the later time of 48 h (Fig. 5C), cultures treated with spIKKγ in the presence of either TNF-α or ETO remained free of viable cells, while in those treated with spIKKγ alone, viable cells remained. In contrast, similar experiments conducted with DG75 cells (a PEL cell line negative for both KSHV and EBV [Fig. 5D]) showed no change in sensitivity to spIKKγ alone and when combined with either TNF-α or ETO. Considerable sensitivity to ETO, however, was detected after 48 h (Fig. 5E), consistent with its use as a therapeutic in the treatment of Burkitt's lymphoma. The effects of SPIKKγ were also investigated on BCP-1 cells, an additional PEL cell line. Similar to results for BC3 cells, significant killing was observed in the presence of peptide alone, but when the peptide was combined with either ETO or TNF-α, none remained viable (Fig. 5F).

## DISCUSSION

The canonical NF-κB pathway is an essential transcriptional mechanism that is hijacked by several viruses to prolong cellular survival and prevent apoptosis, thus producing an environment conducive to viral propagation and ultimately proliferation. Although it has been established that ks-vFLIP is required for protection against apoptosis, in which it constitutively activates the pathway through physical association with IKKγ, it was unclear whether there might also be coinvolvement of the DISC and/or autophagy. Our studies reveal that this protection is mostly independent of both but, interestingly, demonstrates marked differences between the viabilities of ETO-treated Jurkat cells expressing ks-vFLIP in the ATG3 and FADD knockdowns and those treated with TNF-α. It has been reported that autophagy is a specific regulator of homologous recombination (33). This, coupled with the fact that ETO functions by generating double-strand breaks, may explain why rescue, though significant, is more limited in this background. Since ETO is currently in use for the treatment of B cell malignancies, our studies suggest avenues for future chemotherapeutic combinations.

Although the canonical NF-κB pathway itself is an obvious target for the treatment of PEL and other KS-associated malignancies, its requirement in processes such as cellular differentiation, innate/adaptive immunity, and developmental switching (34) make direct drug targeting likely to be associated with considerable toxicity. Given that ks-vFLIP is virally encoded and significantly distinct from its cellular cFLIP homologues both structurally and functionally in terms of its mode of NF-κB activation (18), inhibitors aimed at targeting its interaction with IKKγ are unlikely to have these effects. Here we demonstrate that spIKKγ, a conformationally constrained 20-mer helical peptide comprising the IKKγ interacting element, or an equivalent GST-peptide fusion protein (GST-IKKγ) can interact with ks-vFLIP and disrupt its association with IKKγ. Our crystal structure of ks-vFLIP bound to spIKKγ reveals that its mode of interaction directly mimics that of IKKγ in the original complex to the extent of forming a heterotetrameric ks-vFLIP–spIKKγ assembly with conserved interactions in the spIKKγ-spIKKγ interface. A high-affinity interaction is further illustrated by ITC studies which demonstrate that spIKKγ and IKKγ(150–272) have nearly equivalent binding affinities. We also show that spIKKγ and GST-IKKγ are potent attenuators of ks-vFLIP-induced NF-κB activation, illustrated by *in vitro* studies that include a kinase assay in which the levels of phosphorylated IκB following activation were assessed. Although the effects of vFLIP stimulation appear to be minimal, we find that the basal level of IKK activity in many transformed cell lines is high and that the extent of vFLIP stimulation is correspondingly small, though persistent (18). We have also found that more pronounced effects are not observed when downstream events such as NF-κB transcriptional activity are alternatively assayed.

Although spIKKγ is able to directly instigate BCP-1 and BC3 cell killing, albeit at moderate concentrations (IC_50_ ∼75 μM in BC3 cells, most likely as a consequence of suboptimal permeability based on its comparable affinity to IKKγ), both GST-IKKγ and spIKKγ sensitize PEL to ETO and TNF-α when ks-vFLIP is expressed.

While forming a useful tool compound, spIKKγ provides a template for the production of high-affinity therapeutic peptides given that no attempts were made to optimize the sequence for maximal ks-vFLIP binding or cell permeability. Over the past decade, the use of conformationally constrained helical peptides to target previously intractable protein-protein interactions central to a diverse range of pathologies has grown substantially owing to the ease with which they can be produced, their inherent resistance to proteases, and the fact that they are expected to be well tolerated given their more cognate nature (22, 35). In keeping with this, phase 1 clinical trials of a long-acting growth hormone-releasing hormone agonist peptide for the potential treatment of metabolic/endocrine diseases as well as HIV have so far been successful. In addition to drug-based therapeutics, our studies further illustrate that it is possible to mimic IKKγ using a GST-peptide fusion protein and thus provide an alternative platform for the specific development of PEL- or KS-directed gene therapies.

## MATERIALS AND METHODS

### Cloning, protein expression, and purification. (i) GB1-tagged ks-vFLIP.

ks-vFLIP (residues 1 to 188) was PCR cloned into the EcoRI and XhoI sites of GB1-pBR22b (a modified pET22b vector containing the streptococcal protein G immunoglobulin domain fusion tag [GB1], a kind gift from Paul Driscoll, Francis Crick Institute) using the primers GB1vFLIP-Fwd and GB1vFLIP-Rev (Table 1). The resulting plasmid, designed to generate a ks-vFLIP fusion protein with N- and C-terminal GB1 and 6His tags, respectively (GB1-ks-vFLIP), was transformed into BL21(DE3) cells, for which single colonies were inoculated into LB medium (supplemented with 100 μg ml^−1^ of ampicillin) and grown overnight at 37°C. These cultures were used to inoculate 2-liter flasks containing 800 ml of 100-μg ml^−1^ ampicillin-supplemented LB medium and grown to an optical density at 600 nm [OD_600_] of 0.6 to 0.8. The flasks were subsequently induced with 1 mM isopropyl-β-d-thiogalactopyranoside (IPTG), and the temperature was reduced to 25°C overnight. The cultures were spun at 5,000 × *g* for 20 min and pellets stored at −80°C or resuspended in lysis buffer (20 mM Tris-HCl [pH 8.5] and 200 mM NaCl [buffer A] plus 25 mM imidazole) and subjected to two rounds of lysis using a Vibra cell sonicator. The resulting lysates were spun at 46,000 × *g* and supernatants passed through a 1.2-μm filter prior to being loaded onto a 5-ml His-trap column preequilibrated in buffer A. The column was washed with 20 column volumes (CV) of buffer A and GB1–ks-vFLIP eluted in buffer B (buffer A plus 500 mM imidazole) using a 5-CV step gradient. The resulting protein was then concentrated in a 10-kDa-cutoff Vivaspin concentrator and applied to a Superdex 26/60 S75 size exclusion column preequilibrated with buffer A supplemented with 250 mM imidazole and 5 mM dithiothreitol (DTT) {or 0.3 mM Tris(2-carboxyethylphosphine hydrochloride) [TCEP]}. The eluted fractions were analyzed using SDS-PAGE, and those found to contain GB1–ks-vFLIP at a purity of >95% were pooled, concentrated to 100 μM using a 10-kDa-cutoff Vivaspin concentrator, and stored at 4°C. Fractions used for crystallization trials were dialyzed into a buffer comprising 20 mM Tris-HCl (pH 8.5), 50 mM glycine, and 10 mM TCEP-HCl prior to setup.

**TABLE 1:** List of all primers used to produce the various plasmid constructs detailed together with target sequences for the FADD and ATG3 siRNAs^*a*^

Primer or target	Sequence
GB1vFLIP-Fwd	TTgaattcGATGGCCACTTACG
GB1vFLIP-Rev	AActcgagCGCCTATGGTG
GST Fwd	agatctGCCACCATGTCCCCTATACTAGGTTATTGG
GST Rev	gtcgacctactactaggatccACGCGGAACCAGACCACCACC
GST-IKKγ-Fwd	gatccTTGCAGGTGGCCTATCACCAGCTCTTCCAAGAATACGACAACCACATCAAGAGCAGCGTGGTGGGCAGTtag
GST-IKKγ-Rev	tcgactaACTGCCCACCACGCTGCTCTTGATGTGGTTGTCGTATTCTTGGAAGAGCTGGTGATAGGCCACCTGCAAg
GST-IKKγDM-Fwd	gatccTTGCAGGTGGCCTATCACCAGCTCcgCCAAGAATACcgCAACCACATCAAGAGCAGCGTGGTGGGCAGTtag
GST-IKKγDM-Rev	tcgactaACTGCCCACCACGCTGCTCTTGATGTGGTTGcgGTATTCTTGGcgGAGCTGGTGATAGGCCACCTGCAAg
FADD target 1	CATGGAACTCAGACGCATCTA
FADD target 2	TGAACTCAAGCTGCGTTTATT
ATG3 target 1	CCAGAACTTATGACCTTTACA
ATG3 target 2	ATGTGACCATTGACCATATTT

a The restriction sites, stop codons, and positions mutated in the IKKγ double mutant GST constructs (GST-IKKγDM-Fwd and GST-IKKγDM-Rev) are shown in lowercase and are underlined.

### (ii) IKKγ(150–272) and ks-vFLIP(1–178).

Details for the production and purification of IKKγ(150–272) and ks-vFLIP(1–178) have been previously reported by Bagnéris et al. (19).

### (iii) Expression of GST fusion peptides.

In order to express the IKKγ fusion peptides in Jurkat cells, a GST fusion protein was produced by first PCR amplifying the GST coding region of pGEX-4T1 using Phusion high-fidelity polymerase (New England BioLabs [NEB]) and the primers GST-Fwd and GST-Rev (Table 1). The PCR product was then cloned into a pJET-C1 vector (Fermentas). Following digestion of pJET-C1-GST with BglII and SalI to release the GST coding sequence, the GST product was cloned into the pDual-Cherry lentiviral vector (36) downstream of the spleen focus-forming virus (SFFV) promoter using the BglII/SalI sites. The resulting construct incorporated BamHI and SalI sites at the C terminus of GST that were used for insertion of the peptides. To produce the GST-IKKγ and GST-IKKγDM constructs, IKKγ wild-type peptide primers (GST-IKKγ-Fwd and GST-IKKγ-Rev [Table 1]) corresponding to the spIKKγ protein sequence (residues 230 to 248, including a nonnative C-terminal cysteine) or the same sequence modified to incorporate the F238R D242R double mutant (GST-IKKγDM-Fwd and GST-IKKγDM-Rev), shown to drastically impair ks-vFLIP binding (27), were subsequently annealed at 95°C for 10 min before being ligated into the pDUAL-GST vector following digestion of the vector with BamHI and SalI.

Lentiviral vectors expressing GST or GST fusion peptides together with green fluorescent protein (GFP), CherryFP, WT vFLIP, or vFLIPA57L (a mutant which fails to bind IKKγ) were produced as previously described (19). These were used to transfect unmodified Jurkat cells, those lacking IKKγ (37), those in which ATG3 or FADD was downregulated using a combination of two siRNAs for each mRNA (17) (target sequences provided in Table 1), and also the human B cell line BC3 (a PEL cell line harboring KSHV). In each case, lentiviral vector doses were used to ensure that over 95% of cells were positive for the relevant fluorescent protein. The anti-FADD antibody used for immunoblots was purchased from BD Biosciences, and those for anti-ATG3 and anti-glyceraldehyde-3-phosphate dehydrogenase (anti-GAPDH) were from Cell Signaling Technology. Jurkat cells and their IKKγ-null derivatives were obtained from Alain Israel, Institute Pasteur, and verified as human T cells by surface CD3 staining.

BC3, BCP-1, and DG75 cells were purchased for this study from the American Type Culture Collection (ATCC). They were verified as human B cell lines by surface staining for human Ig.

### Cell viability assays.

The transfected cells, the human B cell lines BC3, BCP-1, and DG75 (a PEL cell line negative for KSHV and Epstein-Barr virus [EBV]) were treated with human TNF-α (PeproTech or Alomone Labs), ETO (Sigma), spIKKγ (encompassing IKKγ residues 230 to 248; see Fig. 3 for structure), or combinations of spIKKγ with either TNF-α or ETO. Cell survival was determined after 24 and 48 h by trypan blue staining and counting with a hemocytometer. Cell viability is expressed as a percentage of the number of viable cells per culture at the start of each experiment. Results show the means and standard deviations of triplicate independent experiments. Significance difference was calculated by two-way analysis of variance (ANOVA) with Fisher's least significant difference (LSD) posttest.

### Thermofluor assays.

Thermofluor studies were conducted using the MyIQ reverse transcription-PCR (RT-PCR) system (Bio-Rad) in a 96-well plate format. Trials were performed in which 30 μM ks-vFLIP (unless otherwise stated) was combined with either IKKγ or mimetic peptides (at the specified concentrations) prior to the addition of SYPRO orange diluted in a 1:1,000 (vol/vol) ratio and thermal denaturation. The data were subsequently processed using the Bio-Rad iQ5 software. Each experiment was repeated at least 3 times. All peptides were purchased from Eurogentec.

### Crystallization and structure determination of the GB1–ks-vFLIP–spIKKγ complex.

spIKKγ (Eurogentec) was first dissolved in 100% dimethyl sulfoxide (DMSO) to a concentration of 2 mM. A 1:1 ratio of GB1–ks-vFLIP to spIKKγ was next combined to give a final GB1–ks-vFLIP concentration of 50 to 70 μM. A Mosquito crystallization robot was used to set up vapor diffusion crystallization trials involving a range of commercially available screens. Drops of 200 nl (final volume) were dispensed in a 1:1 ratio of complex to well solution, and after 1 week, small crystals were observed under the condition 1.2 M ammonium sulfate, 0.05 M trisodium citrate, and 3% isopropanol. In attempts to improve their size and diffraction quality, the Silver Bullet additive screen (Hampton Research) was used in conjunction with the above-described condition. Of the various additives, vitamin B_12_ produced the most significant improvements and was subsequently included in repeat trials of the original condition at a concentration of 0.1 to 0.2%.

Crystals of the GB1–ks-vFLIP–spIKKγ complex, grown in the presence of vitamin B_12_, were cryoprotected in 25 to 30% glycerol and flash frozen in liquid nitrogen prior to irradiation at SOLEIL on the Proxima 1 beamline. Data were collected to a maximum resolution of 3.3 Å on two crystals that were integrated and scaled using XDS (38). The data sets were merged and truncated using AIMLESS (CCP4 [39, 40]) in space group P6_3_. At this stage, the data were found to be significantly anisotropic, which was subsequently corrected for using STARANISO (Global Phasing [http://staraniso.globalphasing.org/cgi-bin/staraniso.cgi]). The effective resolution of the corrected data was reduced to 3.38 Å (giving an overall completeness of >95% [Table 2]). The structure was determined using molecular replacement in PHASER (41) where two GB1–ks-vFLIP monomers were identified in the asymmetric unit. Clear density for peptides associated with each monomer could be observed in the IKKγ binding sites, which were subsequently modeled using COOT (42), along with those of the GB1 monomers which were comparatively poorly ordered. Although side chains could be identified for the majority of residues within each of the spIKKγ peptides, the aliphatic staples were only partially ordered. Refinement was performed using AUTOBUSTER (43), which incorporated noncrystallographic symmetry (NCS) restraints together with translation, libration, screw (TLS) refinement, interspersed with manual rebuilding. Data collection statistics and those for the refined model can be found in Table 2 and are within the expected ranges for a structure at this resolution.

**TABLE 2:** Data collection, processing, and refinement statistics

Parameter	Value(s)^*a*^
Data collection statistics	
Space group	P6_3_
Unit cell [a, b, c (Å)]	a = b = 90.48, c = 134.75, α = β = 90, γ = 120
Resolution (Å)	78.5–3.3 (3.3–3.6)
Total no. of reflections	89,313
No. of unique reflections	9,482
Redundancy	9.4 (9.6)
Completeness (%)	99.80 (99.7)
〈*I*〉/〈σ(*I*)〉	10.3 (1.3)
*R*_meas_^*b*^	0.11 (1.8)
Refinement (STARANISO-corrected data)	
Resolution (Å)	25–3.4 (3.4–3.8)
No. of protein/peptide atoms	3,437
Completeness (%)	95.7 (85.2)
*R*_work_^*c*^/*R*_free_^*d*^ (%)	25.7/29.0
Estimated coordinate error based on *R*_free_ (Å)	0.61
Mean B-factor (Å^2^)	135
Deviations from ideal stereochemistry	
RMSD bonds (Å)	0.009
RMSD angles (°)	1.03
Wilson B-factor (Å^2^)	115
Ramachandran plot analysis^*e*^	
Most favored (%)	95.6
Additionally allowed (%)	2.64
Disallowed (%)	1.76

a Values in parentheses are for the highest-resolution shell (3.4–3.8 Å).

b *R*_meas_ = Σ[(*N*/*N* − 1)]^1/2^[(|Ii − 〈*I*〉|)/Σ(〈*I*〉)], where the sum is calculated over all observations of a measured reflection (Ii), 〈*I*〉 is the mean intensity of all the measured observations (Ii), and *N* is the total number of observations for each reflection.

c *R*_work_ = Σ (|*F*_obs_ − *F*_calc_|)/Σ (*F*_obs_), where *F*_obs_ is the observed structure factor amplitude and *F*_calc_ those calculated from the model.

d *R*_free_ is equivalent to *R*_work_ but where 5% of the measured reflections have been excluded from refinement and set aside for cross-validation purposes.

e Ramachandran plot analysis was from molprobity (http://molprobity.biochem.duke.edu/).

### ITC.

Isothermal calorimetry (ITC) experiments were performed using a Microcal VP-ITC micocalorimeter (MicroCal). For all binding reactions, syringe concentrations varied between ∼85 and 100 μM for IKKγ(150–272) and cell concentrations between ∼8 and 10 μM for GB1-vFLIP. Titrations consisted of an initial 2-μl injection, followed by 20 10-μl injections with stirring at 300 rpm. In competition assays, GB1–ks-vFLIP was first incubated with 20 μM spIKKγ and DMSO to 4% prior to loading into the cell and titration with IKKγ(150–272). All ITC binding experiments were performed in deionized water at 25°C owing to the buffer constraints of spIKKγ, GB1–ks-vFLIP, and IKKγ(150–272), where GB1–ks-vFLIP and IKKγ(150–272) were buffer matched by overnight dialysis, followed by the addition of 4% DMSO to each. It was confirmed that the use of deionized water and 4% DMSO had no discernible impact on structural integrity and/or the affinity of IKKγ(150–272) for GB1–ks-vFLIP by parallel ITC experiments in which both proteins were alternatively dialyzed into a buffer comprising 20 mM Tris-HCl (pH 8.5), 200 mM NaCl, and 0.3 mM TCEP. *K_d_* values comparable to those obtained using deionized water were observed (see “SpIKKγ is an effective mimetic of IKKγ” above and Fig. 4). Following collection, all data were analyzed using ORIGIN 70 software and fitted to a one-site binding model with each experiment performed at least in triplicate.

### Cell-based immunoprecipitation assays.

To probe the ks-vFLIP–IKKγ interaction, Jurkat or BC3 cells were lysed and immunoprecipitation was performed using an anti-IKKγ antibody (FL-419; Santa Cruz Biotechnology) or normal rabbit serum as a control. Lysates and immunoprecipitates were blotted using anti-ks-vFLIP antibodies (6). *In vitro* IKK kinase assays were performed as previously described (17).

### Accession number(s).

The coordinates determined in this study have been deposited at the Protein Data Bank under accession code 5LDE.
